# Application of Near-Infrared Hyperspectral Imaging with Machine Learning Methods to Identify Geographical Origins of Dry Narrow-Leaved Oleaster (*Elaeagnus angustifolia*) Fruits

**DOI:** 10.3390/foods8120620

**Published:** 2019-11-27

**Authors:** Pan Gao, Wei Xu, Tianying Yan, Chu Zhang, Xin Lv, Yong He

**Affiliations:** 1 College of Information Science and Technology, Shihezi University, Shihezi 832000, China; gp_inf@shzu.edu.cn (P.G.); yantianying@163.com (T.Y.); 2 Key Laboratory of Oasis Ecology Agriculture, Shihezi University, Shihezi 832003, China; lxshz@126.com; 3 College of Agriculture, Shihezi University, Shihezi 832003, China; xu_wei082@163.com; 4 Xinjiang Production and Construction Corps Key Laboratory of Special Fruits and Vegetables Cultivation Physiology and Germplasm Resources Utilization, Shihezi 832003, China; 5 College of Biosystems Engineering and Food Science, Zhejiang University, Hangzhou 310058, China; chuzh@zju.edu.cn; 6 Key Laboratory of Spectroscopy Sensing, Ministry of Agriculture and Rural Affairs, Hangzhou 310058, China

**Keywords:** narrow-leaved oleaster fruits, near-infrared hyperspectral imaging, geographical origin, convolutional neural network, effective wavelengths

## Abstract

Narrow-leaved oleaster (*Elaeagnus angustifolia*) fruit is a kind of natural product used as food and traditional medicine. Narrow-leaved oleaster fruits from different geographical origins vary in chemical and physical properties and differ in their nutritional and commercial values. In this study, near-infrared hyperspectral imaging covering the spectral range of 874–1734 nm was used to identify the geographical origins of dry narrow-leaved oleaster fruits with machine learning methods. Average spectra of each single narrow-leaved oleaster fruit were extracted. Second derivative spectra were used to identify effective wavelengths. Partial least squares discriminant analysis (PLS-DA) and support vector machine (SVM) were used to build discriminant models for geographical origin identification using full spectra and effective wavelengths. In addition, deep convolutional neural network (CNN) models were built using full spectra and effective wavelengths. Good classification performances were obtained by these three models using full spectra and effective wavelengths, with classification accuracy of the calibration, validation, and prediction set all over 90%. Models using effective wavelengths obtained close results to models using full spectra. The performances of the PLS-DA, SVM, and CNN models were close. The overall results illustrated that near-infrared hyperspectral imaging coupled with machine learning could be used to trace geographical origins of dry narrow-leaved oleaster fruits.

## 1. Introduction

Narrow-leaved oleaster (*Elaeagnus angustifolia*) is a shrub-like plant of Elaeagnus, which is widely distributed from the Mediterranean region to the northern hemisphere, including in northern Russia and northwestern China. Narrow-leaved oleaster fruits contain a variety of functional health components; in particular, they contain polysaccharides, phenolic acids, and flavonoids. Therefore, narrow-leaved oleaster fruits, as a traditional medicine, are used to treat many diseases in nations and countries from Central Asia to West Asia. As a medicine and food, the fruit of narrow-leaved oleaster fruits is not only a raw material for food industry processing but also a raw material for functional food and new drugs [1,2,3,4,5,6,7,8,9,10,11]. It has good prospects for development and utilization in arid and semi-arid regions of Northwest China. Its unique habitat environment and long history of planting have produced unique qualities of narrow-leaved oleaster fruits in different producing areas. The qualities of narrow-leaved oleaster fruits are different depending on their place of origin, so it is urgent to establish effective methods for identification of the place of origin of narrow-leaved oleaster fruits.

At present, different scholars have isolated the bioactive components of narrow-leaved oleaster fruits [12], studied the physical and chemical properties and antioxidant properties of narrow-leaved oleaster fruits [13], used Gas Chromatography-Mass Spectrometer (GC-MS) to analyze the components of narrow-leaved oleaster fruit oil [14], and studied the diseases of narrow-leaved oleaster fruits [15]. However, there have been few studies on differentiation of the origins of narrow-leaved oleaster fruits. It is feasible to differentiate narrow-leaved oleaster fruits from different producing areas by synthesizing external morphological and microscopic characteristics and physicochemical identification of fruit powder. Manual sorting has many drawbacks, such as involving monotonous work and strong subjectivity, and being time-consuming and difficult to quantify. Physical and chemical index testing is destructive, and requires complicated sample pretreatment, a long detection cycle, and so on. It also has higher professional requirements for testers. These methods are time-consuming and laborious and cannot achieve the goal of fast and non-destructive classification. In view of the drawbacks of traditional detection methods, many applications use hyperspectral imaging for non-destructive detection due to its advantages of non-destructive, rapid, and accurate measurement, which has broad prospects.

Near-infrared hyperspectral imaging is a chemical analysis tool that can detect different absorption frequencies of specific molecules in substances. Near-infrared hyperspectral imaging can acquire spectral and image information of samples simultaneously. It can obtain comprehensive spectral information of samples. It has the characteristics of fastness and high accuracy. Near-infrared hyperspectral imaging has been widely used in geographical origins and variety identification of food [16]. C. Ru et al. used the hyperspectral imaging method of spectral image fusion in the range of visible and near-infrared (VNIR) and shortwave infrared (SWIR) to classify the geographical origin of Rhizoma Atractylodis Macrocephalae [17]. A. Noviyanto et al. used hyperspectral imaging and machine learning to distinguish honey botanical origins [18]. S. Minaei et al. used visible-near-infrared (VIS-NIR) hyperspectral imaging combined with a machine learning algorithm to predict honey floral origins [19]. M. Puneet et al. used near-infrared hyperspectral imaging to identify six different tea products [20]. Our research team has used near-infrared hyperspectral imaging for varietal and geographical origin identification of agricultural and food materials. C. Zhang et al. used near-infrared hyperspectral imaging to identify coffee bean varieties from different locations [21]. W. Yin et al. used near-infrared hyperspectral imaging to identify geographical origins of Chinese wolfberries [22]. S. Zhu et al. used near-infrared hyperspectral imaging to identify cotton seed varieties [23]. These researchers obtained good performances and illustrated the feasibility of using near-infrared hyperspectral imaging to identify the varietal and geographical origin of agricultural and food materials.

In this study, a near-infrared hyperspectral imaging system covering the spectral range of 874–1734 nm was used. This spectral range is related to various chemical compounds. Researchers have used hyperspectral imaging at this spectral range to obtain good performances for determining contents of protein [24], oil [25], water [26], total iron-reactive phenolics, anthocyanins and tannins [27], and flavanol [28], etc. Previous studies have shown that near-infrared hyperspectral imaging can achieve target classification, but there is no relevant research on the place of origin classification of dry narrow-leaved oleaster fruits. The main purpose of this study was to detect the geographical origin of dry narrow-leaved oleaster fruits based on near-infrared hyperspectral imaging technology, combined with characteristic wavelength selection and machine learning algorithms, including deep learning, providing theoretical methods and a basis for distinguishing the different producing areas of narrow-leaved oleaster fruits.

## 2. Materials and Methods

### 2.1. Sample Preparation

**Figure 1:**
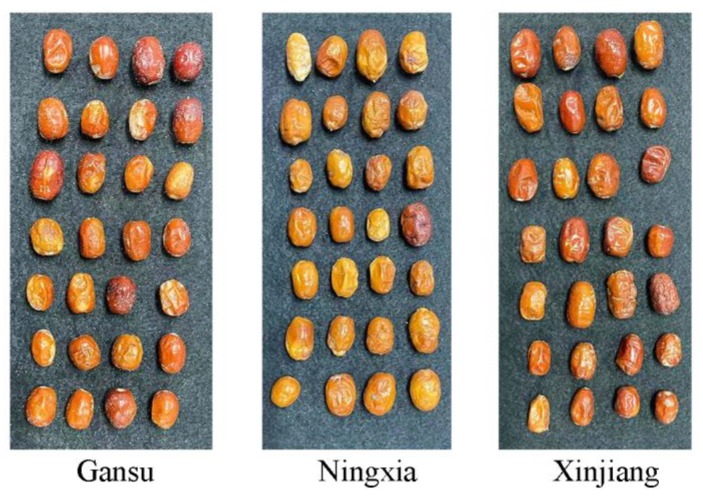
Samples of each geographical origin for hyperspectral imaging acquisition.

Dry narrow-leaved oleaster fruits from three different geographical origins, including Miqin County, Gansu province (Gansu), China (103°4′48″ E, 38°37′12″ N); Zhongwei City, Ningxia Hui Autonomous Region (Ningxia), China (105°10′48″ E, 37°30′36″ N); and Aksu City, Xinjiang Uygur Autonomous Region (Xinjiang), China (80°17′24″ E, 41°9′00″ N), were collected. For each geographical origin, fully matured fruits were harvested in October 2018 and air-dried for consumption and trade. For each geographical origin, intact, clean, and dry narrow-leaved oleaster fruits were collected for hyperspectral image acquisition. In total, 1105, 1205, and 962 intact fruits were obtained from Gansu, Ningxia, and Xinjiang, respectively. The convolutional neural network (CNN) was trained with an independent validation set. To build discriminant models, the samples were randomly split into calibration, validation, and prediction sets. There were 539, 602, and 481 samples from Gansu, Ningxia, and Xinjiang in the calibration set, 291, 303, and 241 samples from Gansu, Ningxia, and Xinjiang in the validation set, and 275, 300, and 240 samples from Gansu, Ningxia, and Xinjiang in the prediction set, respectively. Samples of each geographical origin for hyperspectral imaging acquisition are placed and presented in Figure 1.

### 2.2. Hyperspectral Image Acquisition and Correction

A near-infrared hyperspectral imaging system was used to acquire hyperspectral images of single narrow-leaved oleaster fruits. This hyperspectral imaging system consisted of four major modules, including an imaging module, an illumination module, a sample motion module, and a software module. The imaging module consisted of an imaging spectrograph (ImSpector N17E, Spectral Imaging Ltd., Oulu, Finland) coupled with an InGaAs camera (Xeva 992, Xenics Infrared Solutions, Leuven, Belgium). The spectral range of the hyperspectral imaging system was 874–1734 nm, the spectral resolution 5 nm, and the number of wavebands 256. The lens for the camera was OLES22 (Spectral Imaging Ltd., Oulu, Finland). The illumination module had a 3900 light source (Illumination Technologies Inc., New York, NY, USA). The sample motion module was formed by an IRCP0076 electric displacement table (Isuzu Optics Corp., Taiwan, China) and samples were placed in the motion platform for line-scan. The software module was used to control the image acquisition and motion platform. The structure of the acquired hyperspectral image was able to be expressed as 320 pixels × L pixels × 256 (wavebands), where 320 pixels was the width of the image, the number 256 was the number of wavebands, and L pixels was the length of the image. L was manually determined during the image acquisition to ensure all samples in one plate were covered in one image.

The image quality, which was determined by the distance between the sample and the lens, the moving speed of the motion platform, and the camera exposure time, was determined by setting these parameters as 12.6 cm, 11 mm/s, and 3000 μs, respectively. In this study, intact narrow-leaved oleaster fruits were placed separately on a black plate for image acquisition. For each image, a random number of fruits was placed there (as shown in Figure 1), and there were at least twenty fruits in an image. During image acquisition, the imaging conditions and system parameters always remained. After image acquisition, the raw hyperspectral images were corrected into reflectance images according to the equation

(1) $$I_{c}=\frac{I_{r}-I_{d}}{I_{w}-I_{d}},$$

where *I_c_* is the corrected image, *I_r_* is the raw original image, *I_d_* is the dark reference image and *I_w_* is the white reference image.

### 2.3. Spectral Data Extraction

After image correction, spectral data were extracted from each narrow-leaved oleaster fruit. The hyperspectral imaging system collected reflectance spectra of the samples, and reflectance spectra were used for analysis in this study. Each single narrow-leaved oleaster fruit was defined as a region of interest (ROI). A binary image was formed of each hyperspectral image by binarizing the gray-scale image at 1119 nm, in which the narrow-leaved oleaster fruits region was ‘1’ and the background region was ‘0’. The binary image was then applied to the gray-scale images at each gray-scale image to remove background information. Considering that obvious noises existed at the beginning and end of the spectra, only spectra in the range 975–1646 nm (waveband numbers 31 to 230) were studied, resulting in 200 wavelength variables in the spectral range. Pixel-wise spectra were preprocessed by wavelet transform (wavelet function Daubechies 6 with decomposition level 3) to reduce random noise and area normalization to reduce the influence of sample shape. Pixel-wise spectra within one narrow-leaved oleaster fruit were averaged to represent the sample.

### 2.4. Data Analysis Methods

#### 2.4.1. Principal Component Analysis

Principal component analysis (PCA) is a widely used qualitative analysis and feature extraction method for spectral data analysis. PCA projects the original spectral data to some new principal component variables (PCs) through linear transformation. Each principal component is linearly combined with the original data. The PCs are ranked by the explained variance. The first PC (PC1) explains the largest of the total variance, followed by PC2 and PC3 and so on. In general, the first few PCs could explain most of the total variance and these few principal components with the largest variance could reflect the data information. In general, the scores of scatter plots which are obtained by projecting scores of one PC onto another PC are used to explore clusters of samples from different classes. In this study, PCA was used to explore qualitative discrimination of narrow-leaved oleaster fruit samples from Gansu, Ningxia, and Xinjiang.

#### 2.4.2. Partial Least Squares Discriminant Analysis

The partial least squares discriminant analysis (PLS-DA) algorithm is based on the PLS regression model to discriminate the target, where the variables in the X block (spectral data) are related to the category values corresponding to the classes contained in the Y vector [29,30,31,32,33,34,35]. The integer values are assigned to each class. The category values can be assigned as real integer numbers or they can be formed by dummy variables (0 and 1). PLS regression is firstly conducted on X and Y and the decimal prediction results are transformed into category values according to certain rules.

#### 2.4.3. Support Vector Machine

The support vector machine (SVM) system has been widely applied in statistics, especially for classification. The main idea of SVM is to find the most distinguishable hyperplane by maximizing the margin between the closest points in each class [34,35,36,37,38]. By choosing and optimizing parameters such as penalty factor and kernel function, the discriminant model established by small data samples can still produce small errors for independent test sets. In this paper, the parameter penalty coefficient C of SVM model was searched, and the optimum range was 10^−8^ to 10^8^. The kernel function was a radial basis function (RBF) and the searching range of the width of the kernel function (g) was 10^−8^ to 10^8^.

#### 2.4.4. Convolutional Neural Network

The convolutional neural network has been proved as a data processing method with high efficiency and high performance for hyperspectral data analysis due to its ability to aid automatic feature learning [39]. In this study, a simplified CNN architecture based on the model proposed in [40] was designed for narrow-leaved oleaster fruit discrimination.

**Figure 2:**
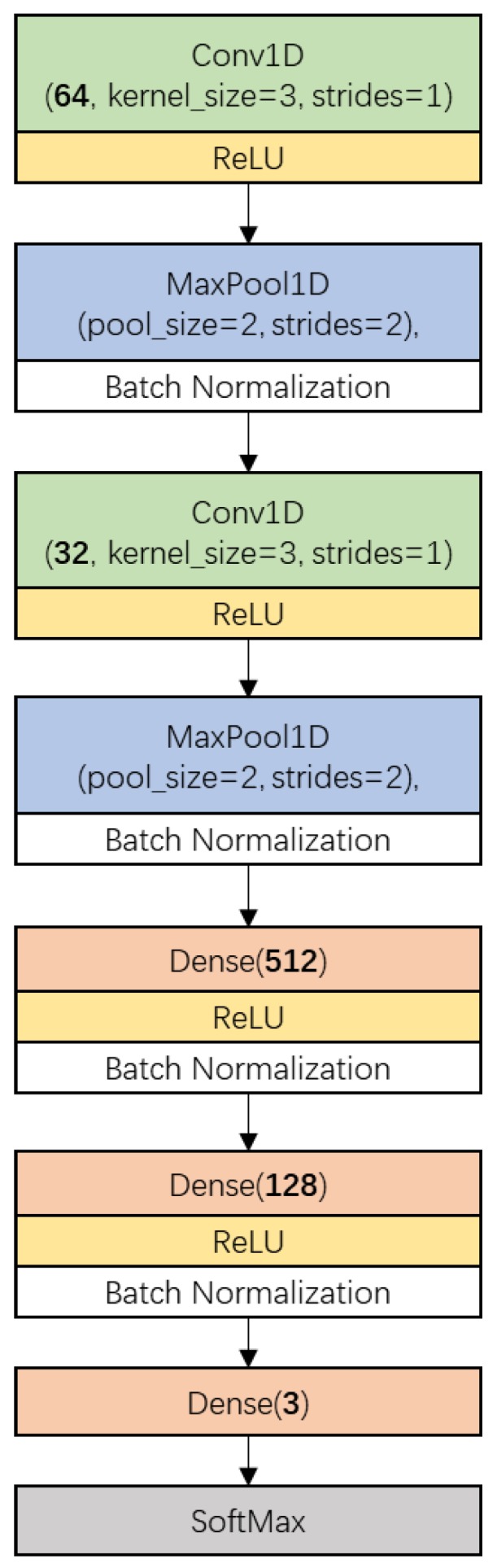
The proposed convolutional neural network (CNN) architecture for narrow-leaved oleaster fruit identification. Conv1D denotes 1-dimension convolution layer, ReLU (Rectified Linear Unit) is the activation function, MaxPool1D denotes 1-dimension max pooling layer, Dense denotes densely-connected neural network layer. The parameter of Conv1D which is defined as ‘Channels’ is the number of the kernels or filters. The parameter of Dense which is defined as ‘units’ is the number of the neurons.

Figure 2 shows the CNN architecture used in this research. It consisted of two main parts. The first part included two one-dimensional convolution layers (Conv1D, represented by a box with a green background), each of which having been followed by a ReLU activation (yellow box), a one-dimension MaxPooling layer (MaxPool1D, blue box) and a batch-normalization (white box) process. The other part included a fully connected network which was constructed by three Dense layers (light red box) and a SoftMax layer (gray box). The numbers of kernels in the convolution layers were 64 and 32, respectively, with a kernel size of 3 and stride of 1 without padding. MaxPooling layers were configured with a pool size of 2 and stride of 2. The numbers of neurons in the Dense layers were defined as 512, 128, and 3, in order. The first two Dense layers were activated by the ReLU function and followed by a batch-normalization process.

The training procedure was implemented by minimizing the SoftMax Cross Entropy Loss using a stochastic gradient descent (SGD) algorithm. The learning rate was optimized and set as 0.0005. The batch size was set as 400. The train epoch was defined as 400.

#### 2.4.5. Optimal Wavelength Selection

Extracted spectra data contain redundant and collinear information, and some of the wavelengths are uninformative. These uninformative wavelengths may result in unstable calibrations. Moreover, a large number of wavelengths for calibration may result in a complex model structure. Selecting the most informative wavelengths is an important step for further multivariate analysis.

In this study, second derivative spectra were used to select the optimal wavelengths for narrow-leaved oleaster fruits. The second derivative is a widely used spectral preprocessing method which can highlight spectral peaks and suppress background information. In second derivative spectra, the background information is quite small and close to zero, and the positive and negative peaks with greater differences among different categories of samples are manually selected as optimal wavelengths [41].

### 2.5. Software and Model Evaluation

In this study, PCA, PLS-DA, and SVM were executed on a Matlab R2014b (The Math Works, Natick, MA, USA), the second derivate was conducted on Unscrambler 10.1 (CAMO AS, Oslo, Norway), and the CNN model was performed on Python 3 and MXNET framework (Amazon, Seattle, WA, USA). PCA and PLS-DA was computed using leave-one-out cross validation, SVM was computed using five-fold cross validation, and CNN was computed using an independent validation set. Model performances were evaluated by their classification accuracy, which was calculated as the ratio of the number of correctly classified samples to the total number of samples.

## 3. Results

### 3.1. Spectral Profiles and Effective Wavelength Identification

**Figure 3:**
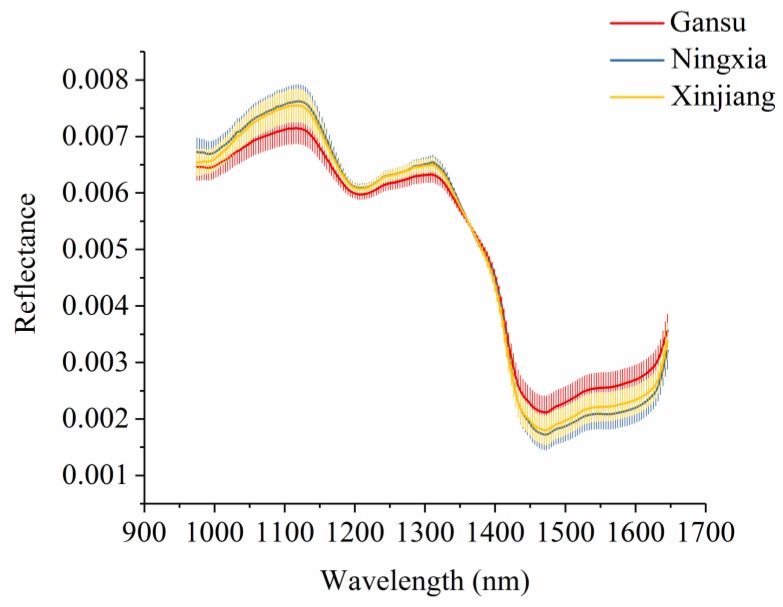
Average spectra with standard deviation of each wavelength of narrow-leaved oleaster fruits from Gansu, Ningxia, and Xinjiang.

**Figure 4:**
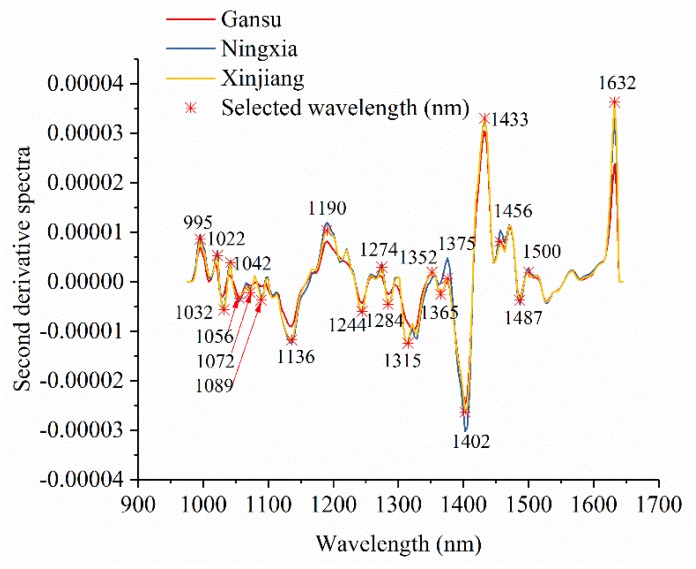
Effective wavelength selection using the second derivative spectra of average spectra of the samples from Gansu, Ningxia, and Xinjiang.

Figure 3 shows the average spectra with standard deviation of each wavelength of narrow-leaved oleaster fruits from Gansu, Ningxia, and Xinjiang. Slight differences in reflectance values exist in the average spectra. The differences exist across the whole spectral ranges. However, the overlaps can be observed according to the standard deviation in Figure 3. With these overlaps, the samples from different geographical origins cannot simply be identified by observing their spectral differences. Figure 4 shows the second derivative spectra of the average spectra of narrow-leaved oleaster fruit samples from Gansu, Ningxia and Xinjiang. There are wavelengths with differences. Wavelengths corresponding to the peaks and valleys with greater differences were manually identified. As shown in Figure 4, a total of 22 wavelengths can be identified: 995, 1022, 1032, 1042, 1056, 1072, 1089, 1136, 1190, 1244, 1274, 1284, 1315, 1352, 1365, 1375, 1402, 1433, 1456, 1487, 1500, and 1632 nm. These wavelengths were selected as the effective wavelengths for geographical identification. In this study, the full spectra were used to conduct PCA for qualitative analysis of the sample cluster within one geographical origin and sample separability among different geographical origins. The full spectra were also used to build machine learning models to quantitatively assess the sample separability among different geographical origins. To reduce redundant and collinear information which are informative in full spectra, simplify the models and improve model robustness, the selected effective wavelengths were used to build machine learning models for comparison with the full-spectra-based models.

### 3.2. Principal Component Analysis

**Figure 5:**
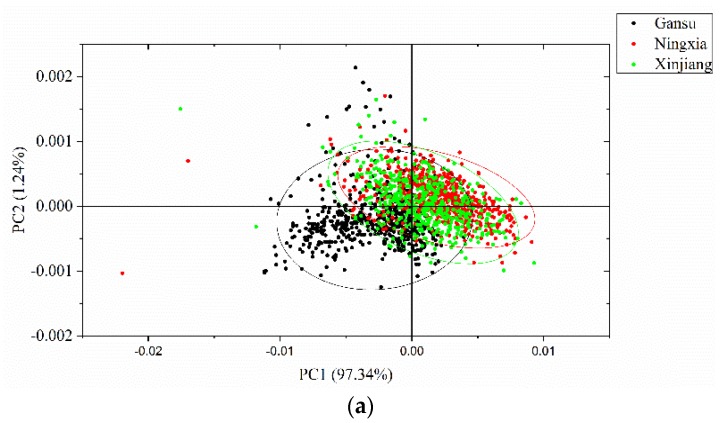

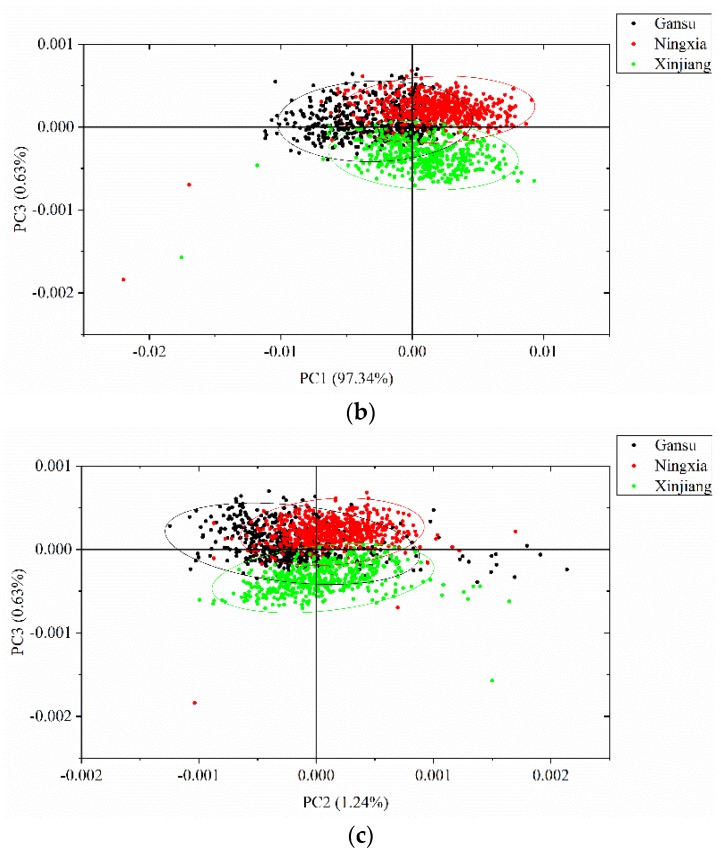
Principal component analysis (PCA) score scatter plots of (**a**) PC1 versus PC2; (**b**) PC1 versus PC3; and (**c**) PC2 versus PC3. The ellipse is the confidence ellipse (confidence level at 0.95).

PCA was conducted to qualitatively cluster the samples in the scoring spaces. PCA was conducted on the full spectra of the calibration set, and the spectral data were centered for PCA analysis. The first three PCs explain most of the total variance, which was over 99% (PC1: 97.34%, PC2: 1.24%, PC3: 0.63%). Score scatter plots of two different PCs are shown in Figure 5. Samples from the same geographical origins are marked with the same color, as well as the confidence ellipse (confidence level at 0.95). As shown in the score scatter plot of PC1 versus PC2, samples from each geographical origin are able to cluster well. Overlaps exist among the samples from Gansu, Ningxia, and Xinjiang. In the score scatter plot of PC1 versus PC3, samples from each geographical origin are able to cluster well. Samples from Gansu show greater overlaps with samples from the other geographical origins, and samples from Ningxia and Xinjiang are able to separate well. In the score scatter plot of PC2 versus PC3, samples from each geographical origin are able to cluster well. Samples from Gansu show greater overlaps with samples from the other geographical origins, and samples from Ningxia and Xinjiang are able to separate well. The score scatter plots in Figure 5 showed that the samples from different geographical origins are able to be well clustered and that they have great potential to be correctly identified.

### 3.3. Classification Models Using Full Spectra

PLS-DA, SVM, and CNN models were built using the full spectra. For the PLS-DA models, the category values of the samples from Gansu, Ningxia, and Xinjiang were labelled 001, 010, and 100. For the SVM and CNN models, the category values of the samples from Gansu, Ningxia, and Xinjiang were labelled 0, 1, and 2.

The classification results of the three different models are shown in Table 1. All discriminant models obtained good performances, with the classification accuracy of the calibration, validation, and prediction sets all over 90%. For the PLS-DA model, the optimal number of latent variables (LVs) was 12, and good classification performance was obtained. Classification accuracies of the calibration, validation, and prediction sets were all over 99%. For the SVM model, the model parameters (C, g) were optimized as (100, 10,000). The classification accuracy of the calibration set was 100%, while the classification accuracy of the validation and prediction sets was found to be lower. For the CNN model, the classification accuracy of the calibration, validation, and prediction sets were determined to be all over 97%. With regard to all three models, the PLS-DA model performed the best, the CNN model obtained results quite close to and slightly worse than those for PLS-DA, and the SVM model performed the worst.

When using the PLS-DA model, samples from Ningxia were misclassified as samples from Xinjiang and samples from Gansu were misclassified as samples from Xinjiang; when using the SVM model, samples from Gansu were misclassified as samples from Xinjiang; and when using the CNN model, samples from Gansu and Xinjiang were misclassified as each other. The overall classification results indicated good separability among the samples from the three geographical origins. Samples from Gansu and Xinjiang were more likely to be misclassified, due to the results of the three discriminant models.

### 3.4. Classification Models Using Optimal Wavelengths

After effective wavelength selection, the PLS-DA, SVM, and CNN models were built using the selected effective wavelengths. The results of the three discriminant models are shown in Table 2. Good performances were obtained by the three models, with the classification accuracy of the calibration, validation, and prediction sets all over 95%. For the PLS-DA model, the optimal number of LVs was found to be 17. The classification accuracies of the calibration, validation, and prediction sets were all over 99%. For the SVM model, the model parameters (C, g) were optimized as (100, 108). The classification accuracies of the calibration, validation, and prediction sets were all over 95%. For the CNN model, the classification accuracies of the calibration, validation, and prediction sets were all over 97%.

When using the PLS-DA model, samples from Gansu and Xinjiang were misclassified as each other, and one sample from Ningxia was misclassified as a sample from Gansu. When using the SVM model, it was observed that samples from Gansu and Xinjiang were misclassified as each other. When using the CNN model, samples from Gansu and Xinjiang were misclassified as each other, and one sample from Ningxia was misclassified as a sample from Xinjiang. The confusion matrices of the three models illustrate that samples from Gansu and Xinjiang were more likely to be misclassified.

The PLS-DA, SVM, and CNN models using effective wavelengths obtained similar results to those using effective wavelengths, illustrating the effectiveness of effective wavelength selection. The overall classification accuracy of all models indicates that there are great differences existing in narrow-leaved oleaster fruits from the three different geographical origins considered. As shown in Table 1 and Table 2, the PLS-DA models performed slightly better than the CNN models, and the CNN models performed slightly better than the SVM models. Although differences existed in these model performances, the differences were quite small. The results illustrate that CNN models could be used for narrow-leaved oleaster fruit geographical origin identification. Moreover, the results of the discriminant models using full spectra and effective wavelengths all showed that samples from Gansu and Xinjiang were more likely to be misclassified.

## 4. Conclusions

In this work, near-infrared hyperspectral imaging was successfully used to identify the geographical origins of narrow-leaved oleaster fruits from Gansu, Ningxia, and Xinjiang. PCA score scatter plots showed the separability of the samples from the three geographical origins. PLS-DA, SVM, and CNN models were established using full spectra and effective wavelengths selected by second derivative spectra. The high classification accuracy, which was over 90% for models using full spectra and effective wavelengths, illustrates that the proposed method can effectively distinguish narrow-leaved oleaster fruits from different geographical origins. The performances of the models using effective wavelengths were similar to those using full spectra. Moreover, deep CNN models obtained close results to the PLS-DA and SVM models, showing good performances of deep learning for narrow-leaved oleaster fruit geographical origin detection. According to the discriminant models, samples from Gansu and Xinjiang were more likely to be misclassified. These results indicate that it would be possible to develop online systems for narrow-leaved oleaster fruit origin detection using near-infrared hyperspectral imaging and machine learning methods.

## Figures and Tables

**Table 1:** Confusion matrix of the partial least squares discriminant analysis (PLS-DA), support vector machine (SVM) and convolutional neural network (CNN) models using full spectra.

Model	Category Values	Calibration				Validation				Prediction			
		0	1	2	Total (%)	0	1	2	Total (%)	0	1	2	Total (%)
**PLS**	0 *	539	0	0		291	0	0		268	0	7	
	**1**	0	601	1		0	303	0		0	299	1	
	**2**	0	0	481		0	0	241		0	0	240	
	**Total (%)**				99.94				100				99.02
**SVM**	**0**	539	0	0		289	0	2		224	0	51	
	**1**	0	602	0		0	303	0		0	300	0	
	**2**	0	0	481		0	0	241		0	0	240	
	**Total (%)**				100				99.76				93.74
**CNN**	**0**	539	0	0		289	0	2		253	0	22	
	**1**	1	601	0		0	303	0		0	300	0	
	**2**	6	0	475		4	0	237		0	0	240	
	**Total (%)**				99.57				99.28				97.30

* 0, 1, and 2 are the assigned category values of the samples from Gansu, Ningxia, and Xinjiang, respectively.

**Table 2:** Confusion matrices of the PLS-DA, SVM, and CNN models using effective wavelengths.

Model	Category Values	Calibration				Validation				Prediction			
		0	1	2	Total (%)	0	1	2	Total (%)	0	1	2	Total (%)
**PLS**	0 *	538	0	1		291	0	0		272	0	3	
	**1**	1	601	0		0	303	0		0	300	0	
	**2**	1	0	480		0	0	241		0	0	240	
	**Total (%)**				99.92				100				99.63
**SVM**	**0**	539	0	0		271	0	20		238	0	37	
	**1**	0	602	0		0	303	0		0	300	0	
	**2**	2	0	479		1	0	240		1	0	239	
	**Total (%)**				99.88				97.49				95.34
**CNN**	**0**	539	0	0		287	0	4		263	0	12	
	**1**	0	602	0		0	303	0		0	299	1	
	**2**	4	0	477		8	0	233		5	0	235	
	**Total (%)**				99.75				98.56				97.79

* 0, 1, and 2 are the assigned category values of the samples from Gansu, Ningxia, and Xinjiang, respectively.
